# Comparison Between the Video Head Impulse Test and Caloric Irrigation During Acute Vertigo

**DOI:** 10.1007/s12070-022-03123-z

**Published:** 2022-08-01

**Authors:** Elin Olivecrona, Katarina Zborayova, Marie-Louise Barrenäs, Jonatan Salzer

**Affiliations:** 1 Clinical Science, Neurosciences, Umeå University, 90187 Umeå, Sweden; 2 Clinical Science, Otorhinolaryngology, Umeå University, Umeå, Sweden

**Keywords:** Vertigo, Dizziness, Caloric irrigation, Video head impulse test

## Abstract

Caloric irrigation (CI) is the gold standard to investigate peripheral vestibular dysfunction. The video head impulse test (vHIT) is faster and more accessible and may be useful during acute vertigo stroke risk differentiation. Comparative studies between the two methods are needed. The objective of this study was to compare vestibular function data derived from caloric irrigation with that from vHIT. This study included 80 patients with acute onset vertigo who underwent caloric irrigation and vHIT. CI derived sum of slow phase velocities (SPVs) and unilateral weakness (UW) were compared with vHIT vestibulo-ocular reflex (VOR) gain and gain asymmetry (GA) using correlation analyses. Optimal cut offs for vHIT VOR gain and GA were calculated using Youden indexes. There was a strong positive correlation between the asymmetry measures UW and GA whereas the correlation between the sum of SPVs and VOR gain was weaker. The optimal cut offs to diagnose unilateral vestibular weakness were 0.80 for VOR gain and 28% for GA; with specificities for predicting normal caloric irrigation results of 55% and 93%, respectively. In one third of cases the results from caloric irrigation and vHIT dissociated. The results from vHIT correlated with those from CI, still neither test seem to have the accuracy to replace the other. GA appears as an attractive measure in acute vertigo as the high specificity can be used to identify those with a substantial probability of normal vestibular function in need of more comprehensive work-up for central causes. To diagnose vestibular dysfunction, CI remains gold standard.

## Introduction

Vertigo is a common cause for seeking health care and constitutes about 3% of the emergency department visits in Sweden in the adult population [1]. The diagnostics of vertigo usually involves testing the vestibular function by measuring the vestibulo-ocular reflex (VOR) and its response to semicircular canal stimulation. Two diagnostic tests available for this purpose are the caloric irrigation and video head impulse test (vHIT). Caloric irrigation originates back to 1906 and is still seen as the gold standard method for diagnosing vestibular hypofunction. It is based on evaluation of the VOR response to ear-specific irrigations with water or air of standardized temperatures by using videonystagmography [2]. Some of the drawbacks of caloric irrigation are that it is time-consuming, unpleasant and the individual responses are variable [3]. The Head Impulse Test (HIT) evaluates the eye-responses of the VOR during rapid head turns with the patient’s eyes simultaneously fixated on a stationary target. Vestibular hypofunction is seen as catch-up saccades after the impulses [4]. The HIT was introduced in 1988 as a simple bedside test (bHIT) and was later improved by adding a video recording system, creating the vHIT. This new system enabled quantitative evaluation of the VOR and detection of eye movements during the head impulses which are imperceptible to the naked eye of the examiner [5].

Previous studies have indicated that there is a correlation between the results of the caloric test and vHIT in chronic dizziness [3, 6–9]. The aims of this study were to investigate, in patients with acute vertigo, the correlations between caloric irrigation and vHIT measures; and to determine the optimal vHIT VOR gain and gain asymmetry (GA) cut offs to detect vestibular hypofunction.

## Materials and Methods

### Study Population

This study included 80 patients seeking healthcare due to acute onset vertigo at Umeå University hospital between May 2015 and January 2019. All patients were investigated as part of a prospective observational diagnostic study on acute vertigo and underwent clinical examination, including bHIT, vHIT, caloric irrigation and radiology. The inclusion criterion for the study was an acute vestibular syndrome, defined as acute onset of a new continuous and ongoing vertigo since less than 72 h with at least two of the four signs or symptoms: pathological nystagmus, nausea/vomiting, balance disturbance or discomfort during head movements. Patients from the prospective cohort (n = 183) were excluded if they had not undergone vHIT or caloric irrigation or if the test results were incomplete. Patients were diagnosed as followed: vestibular neuritis [4], Meniere’s disease [10], vestibular migraine [11], benign paroxysmal positional vertigo [12], stroke/transient ischemic attack [13] and vestibular schwannoma (verified by MR). Patients with a unilateral weakness (UW) > 25% who did not meet the criteria for the diagnoses mentioned above were categorized as “unilateral vestibulopathy of unknown cause” and the remainder as “vertigo not otherwise specified”. Patients were divided into two subgroups based on their caloric response: (1) subjects with vestibular hypofunction (defined as UW > 25% and/or sum of Slow Phase Velocities (SPV) below 15°/s for each vestibular organ) and (2) subjects with normal vestibular function.

### Caloric Irrigation

Caloric irrigations were performed in a supine position with the head of the patient elevated 30°. It was done as a conventional bithermal caloric test flushing first warm (44 °C) and then cold (30 °C) water into the right (R) and left (L) ear while measuring the vestibular nystagmus responses. The irrigations lasted for 30 s for each of the four measurements: R44°, L44°, R30° and L30°. The interval between every irrigation was at least 5 min. A subset of patients with normal responses (defined as UW ≤ 25%) during the warm irrigation did not proceed to receive cold irrigation (n = 28), in accordance with the clinic's routines at the time. A video-based system (Ulmer VNG, Synapsys; Marseille, France) was used to obtain and analyze the nystagmus responses during each irrigation. The maximum SPVs of the nystagmus beat were analyzed and used for calculating UW using Jongkees formula: (R30° + R44°)−﻿(L30° + L44°)/(R30° + R44° + L30° + L44°) × 100 = % UW [7, 14]. For patients with monothermal caloric irrigation, a modified version of the formula was used: (R44°−L44°)/(R44° + L44°) × 100 = % UW [15]. An asymmetry between the nystagmus responses for the right and left ear of more than 25% was considered pathological and classified as unilateral vestibular hypofunction [6, 8]. Bilateral vestibular hypofunction was classified as sum of SPV for irrigation with warm and cold water below 15°/s for each vestibular organ (R44° + R33° and L44° + L33°) [3].

### Bedside Head Impulse Test

The bHIT was performed either by the on-call physician in the emergency room or by an otorhinolaryngology physician. Patients were placed in an upright sitting position facing the clinician and were asked to keep their eyes fixated on the examiners nose. The clinician held the patients head firmly with both hands and turned it rapidly to either the right or left at approximately 10–20° angle. This was repeated a few times in each direction. The patients’ eye movements were closely observed during the head impulses to notice any catch-up saccades, indicating a pathological VOR. The bHIT was considered abnormal if any catch-up saccades were seen with the naked eye of the examiner.

### Video Head Impulse Test

All vHIT tests were performed by one of the two specialized technicians in our vestibular laboratory. The patients were placed in a sitting position and asked to keep their eyes fixated on a stationary target on the wall straight ahead at a distance of 1.9 m. The investigator was standing behind the patient and delivered head impulses with an unpredictable timing and direction. On examination of lateral semicircular canals, the patient’s head was leaned forward at approximately 30° angle and turned laterally during the impulses. The amplitude of each impulse was 10–20°. Head impulses not meeting the software predetermined velocity, acceleration, trajectory, and pupil detection requisites were automatically discarded. Impulses were delivered until at least 5 approved reactions were recorded for each semicircular canal. The eye movements were measured by a floor-mounted vHIT system (Ulmer Synapsys vHIT II, software version 14.1; Marseille, France) standing at a distance of 0.9 m in front of the patient. The VOR gain was calculated by the software using the ratio between mean eye velocity (°/s) to mean head velocity (°/s), measured as the area under the velocity curve for both parameters. A pathological vHIT result was defined as a VOR gain of ≤ 0.8 for the lateral semicircular canal in combination with the presence of pathological eye catch-up saccade(s). Gain asymmetry (GA) was calculated using the formula: GA = (R−L)/(R+L), where R and L represents the mean value of VOR gain for lateral impulses to the right (R) and left (L) side [8].

### Statistical Analyses

Baseline data were presented using descriptive statistics. The sum of SPVs of warm and cold irrigation and corresponding caloric asymmetry measure (UW) were compared with lateral canal vHIT VOR gain and GA using Spearman’s correlation coefficient. In these analyses, UW was converted to a negative value when lateralized to the right, similar to how GA was presented. A *p*-value of < 0.05 was considered statistically significant. The diagnostic accuracies for vHIT and bHIT were evaluated using the caloric irrigation as reference method; sensitivities, specificities, positive predictive values, and negative predictive values with 95% confidence interval (CI) were calculated from four-field tables. Two different receiver-operating characteristics (ROC) curves were drawn to evaluate the diagnostic performance of different cut-offs of vHIT VOR gain and GA based on the results from caloric irrigation. Youden indexes (sensitivity + specificity—1) were calculated to determine the optimal cut-off values for VOR gain and GA. All statistical analyses were performed using SPSS® V26, IBM; New York, USA.

## Results

Out of 183 patients found in the study database in autumn 2019, 80 fulfilled the inclusion criteria and were included in this study. One-hundred and three patients were excluded due to lack of written consent (n = 1), congenital nystagmus (n = 1), missing result from vHIT (n = 8) or caloric irrigation (n = 91) or both (n = 2). The study sample consisted of 44 male and 36 female subjects with an age range 18–92 years, mean age 62 years (Table 1). Bithermal caloric irrigation was performed in 65% (n = 52) of all patients whilst the remaining n = 28 cases underwent the monothermal warm caloric screening test (Table 1). Vestibular hypofunction, as determined by the results from caloric irrigation, was found in 48% (n = 38) (Table 2). Thirty-seven of these patients had unilateral vestibular hypofunction whereas one showed bilateral deficits. The results from both caloric irrigation and vHIT are summarized in Table 3.

**Table 1:** Baseline characteristics of the n = 80 subjects with acute onset vertigo

	All subjects, n = 80	Vestibular hypo-function^a^, n = 38	Normal vestibular function, n = 42
Age (years, ± SD)	62 ± 17	60 ± 15	64 ± 19
*Sex (n, %)*			
Male	44 (55.0)	22 (57.9)	22 (52.4)
Female	36 (45.0)	16 (42.1)	20 (47.6)
*Diagnoses (n, %)*			
Vestibular neuritis	19 (23.8)	19 (50.0)	0 (0.0)
Benign paroxysmal positional vertigo	11 (13.8)	4 (10.5)	7 (16.7)
Meniere’s disease	6 (7.5)	3 (7.9)	3 (7.1)
Stroke/transient ischemic attack	7 (8.8)	2 (5.3)	5 (11.9)
Vestibular schwannoma	1 (1.3)	0 (0.0)	1 (2.4)
Vestibular migraine	1 (1.3)	0 (0.0)	1 (2.4)
Unilateral vestibulopathy of unknown cause	10 (12.5)	10 (26.3)	0 (0.0)
Vertigo not otherwise specified	25 (31.3)	0 (0.0)	25 (59.5)
Caloric irrigation (n, %)			
Bithermal irrigation	52 (65.0)	37 (97.4)	15 (35.7)
Monothermal irrigation 44 °C	28 (35.0)	1 (2.6)	27 (64.3)
Time intervals (mean, ± SD)			
Days from emergency room to bHIT	1.3 ± 1.0	0.97 ± 1.38	1.54 ± 2.65
Days from emergency room to vHIT	1.3 ± 2.2	1.29 ± 1.14	1.21 ± 0.98
Days from emergency room to caloric irrigation	11.6 ± 2.5	13.79 ± 16.26	9.64 ± 7.49

^a^Vestibular hypofunction was defined as unilateral weakness > 25% and/or sum of slow phase velocities < 15°/s per vestibular system. *vHIT* video head impulse test. *bHIT* bedside head impulse test

**Table 2:** Diagnostic rate of the bedside Head Impulse Test and video Head impulse test compared with caloric irrigation in n = 80 subjects with acute onset vertigo

HIT	Caloric irrigation: Abnormal^a^ (n, %)	Normal (n, %)	Sum (n)
*bHIT*			
Abnormal	12 (41)	7 (18)	19
Normal	17 (59)	32 (82)	49
Total	29 (100)	39 (100)	68^b^
*vHIT*			
Abnormal^c^	28 (74)	16 (38)	45
Normal	10 (26)	26 (62)	35
Total	38 (100)	42 (100)	80^d^

^a^Abnormal caloric irrigation defined as unilateral weakness > 25% and/or sum of slow phase velocities < 15°/s per vestibular system. ^b^Sixty-eight patients were included. Patients were excluded from this analysis if they lacked result from bHIT or if their results were impossible to interpret (n = 12)

^c^Abnormal vHIT was defined as VOR gain in the affected lateral canal < 0.8 in combination with pathological catch-up saccades *or* VOR gain in any lateral canal < 0.8 in combination with pathological catch-up saccades

^d^All patients were included

*bHIT* bedside head impulse test, *vHIT* video head impulse test, *VOR* vestibulo-ocular reflex

**Table 3:** Quantitative vestibular vHIT and caloric irrigation data for n = 80 subjects with acute onset vertigo

vHIT	All subjects, n = 80		Vestibular hypofunction^a^, n = 38		Normal vestibular function, n = 42	
	Mean (± SD)	N	Mean (± SD)	N	Mean (± SD)	N
Gain asymmetry (%)	33.7 ± 44.2	80	57.8 ± 51.1	38	11.9 ± 20.0	42
*Right lateral*						
VOR gain	0.72 ± 0.31	80	0.63 ± 0.36	38	0.81 ± 0.25	42
Impulses (N)	5.83 ± 1.68	76	5.83 ± 1.58	36	5.82 ± 1.78	40
Sigma	0.08 ± 0.12	70	0.11 ± 0.17	30	0.07 ± 0.06	40
*Left lateral*						
VOR gain	0.67 ± 0.73	79	0.51 ± 0.42	38	0.82 ± 0.24	41
Impulses (N)	6.18 ± 1.83	76	6.36 ± 1.90	36	6.03 ± 1.78	40
Sigma	0.10 ± 0.11	66	0.13 ± 0.15	27	0.08 ± 0.06	39
VOR gain, affected side^b^	0.58 ± 0.41	80	0.36 ± 0.42	38	0.78 ± 0.28	42
VOR gain, unaffected side	0.82 ± 0.20	80	0.79 ± 0.19	38	0.84 ± 0.20	42
*CALORIC IRRIGATION*						
UW (%)	37.6 ± 36.0	80	67.2 ± 31.8	38	10.9 ± 7.5	42
Total response^c^ (°/s)	70.4 ± 38.4	52	62.8 ± 34.2	37	89.3 ± 43.5	15
SPV right ear 44° (°/s)	26.6 ± 16.2	80	24.5 ± 16.1	38	28.5 ± 16.2	42
SPV right ear 30° (°/s)	13.0 ± 9.0	52	11.6 ± 9.2	37	16.6 ± 7.6	15
Sum of SPV right ear^d^ (°/s)	38.1 ± 23.6	52	35.8 ± 23.3	37	43.8 ± 24.1	15
SPV left ear 44° (°/s)	26.6 ± 38.6	80	17.0 ± 16.6	38	35.3 ± 49.6	15
SPV left ear 30° (°/s)	10.9 ± 9.6	52	9.5 ± 9.3	37	14.2 ± 9.7	15
Sum of SPV left ear^d^ (°/s)	32.3 ± 25.1	52	26.9 ± 24.6	37	45.5 ± 21.8	15
Sum of SPV, affected side^b^ (°/s)	23.1 ± 17.7	52	16.6 ± 12.6	37	39.2 ± 18.7	15

^a^Vestibular hypofunction was defined as unilateral weakness > 25% and/or sum of slow phase velocities < 15°/s per vestibular system

^b^The affected side was defined as the side with the lowest caloric response

^c^Total response = SPV R44° + SPV R30° + SPV L44° + SPV L30°

^d^Sum of SPV = R44° + R33° and L44° + L33°

*vHIT* video head impulse test, *UW* unilateral weakness, *VOR* vestibulo-ocular reflex, *SPV* slow phase velocities

Seven patients were diagnosed with stroke or TIA. The affected vascular territories were distributed as follows: left anterior inferior cerebellar artery (AICA) infarction (n = 3), left posterior inferior cerebellar artery (PICA) infarction (n = 1), lacunar right middle cerebral artery (MCA) infarction (n = 1), vertebral artery TIA (n = 2). Two out of these 7 patients (1 with AICA infarction and 1 with TIA) had vestibular hypofunction (UW > 25%) whereas the others presented with normal vestibular function on caloric irrigation. Pathological results on vHIT were detected in 6 out of 7 of these patients. The patient with normal vHIT had an AICA infarction and normal results on caloric irrigation.

### Correlations Between Caloric Irrigation and vHIT

**Fig. 1:**
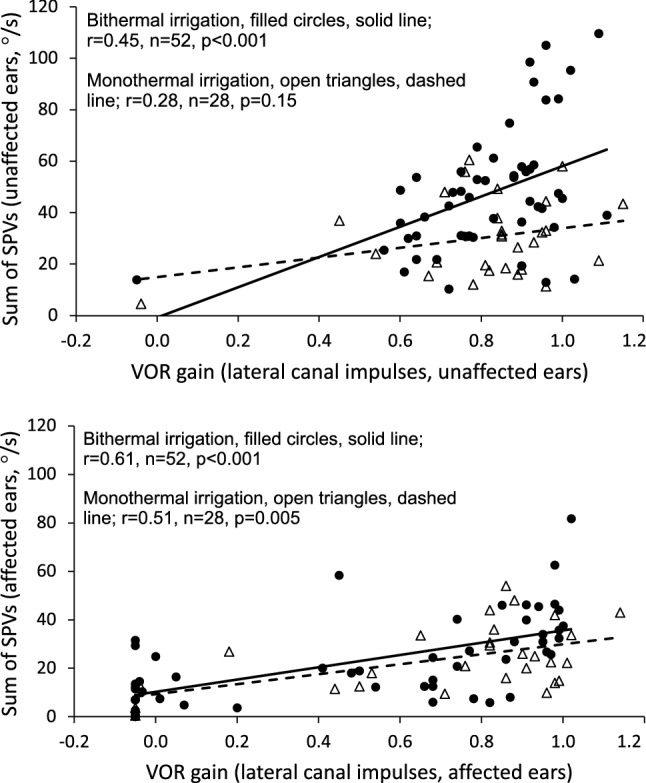
**a** and **b** Scatterplots of the correlation between the sum of SPVs during caloric irrigation and VOR gain on vHIT. **a** Unaffected ears, **b** Affected ears, where the affected ear was defined as the ear with the lowest caloric response. Cases with bithermal caloric irrigation are denoted with filled circles, solid line. Cases with monothermal caloric irrigation are denoted with open triangles, dashed line. SPV, slow phase velocities. VOR, Vestibulo-ocular reflex. vHIT, Video Head Impulse Test

**Fig. 2:**
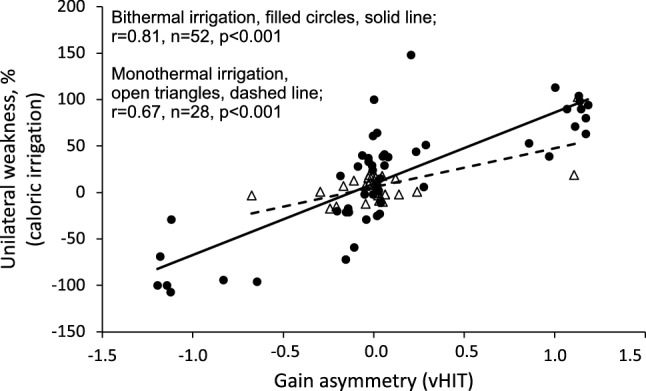
Scatterplot of the correlation between unilateral weakness on caloric irrigation and gain asymmetry on vHIT. Cases with bithermal caloric irrigation are denoted with filled circles, solid line. Cases with monothermal caloric irrigation are denoted with open triangles, dashed line. Both measures were assigned positive values when lateralized to the left and negative values when lateralized to the right. vHIT, video Head Impulse test

Analyses of the correlation between caloric irrigation sum of SPVs and vHIT VOR gain were conducted separately for affected and unaffected ears in all patients. The affected ear was defined as the ear with the lowest caloric response. There were weak to moderate positive correlations in unaffected ears and moderate positive correlations in affected ears (Fig. 1a, b). Positive UW and GA values indicated lateralization to the left and negative values lateralization to the right. There were moderate to strong positive correlations between UW and GA (Fig. 2). In all correlation analyses bithermal caloric irrigation correlated stronger with vHIT compared with monothermal caloric irrigation.

### Diagnostic Value of bHIT and vHIT as Compared to Caloric Irrigation

The diagnostic accuracies of bHIT and vHIT as compared to caloric irrigation are presented in Table 2. The bHIT showed a sensitivity of 41% (95% CI: 24–61%) and a specificity of 82% (95% CI: 67–93%), giving a positive predictive value (PPV) of 63% (95% CI: 44–79%) and a negative predictive value (NPV) of 65% (95% CI: 57–73%). The vHIT displayed a sensitivity of 74% (95% CI: 57–87%) and a specificity of 62% (95% CI: 46–76%), giving a PPV of 64% (95% CI: 53–73%) and a NPV of 72% (95% CI: 59–82%).

**Fig. 3:**
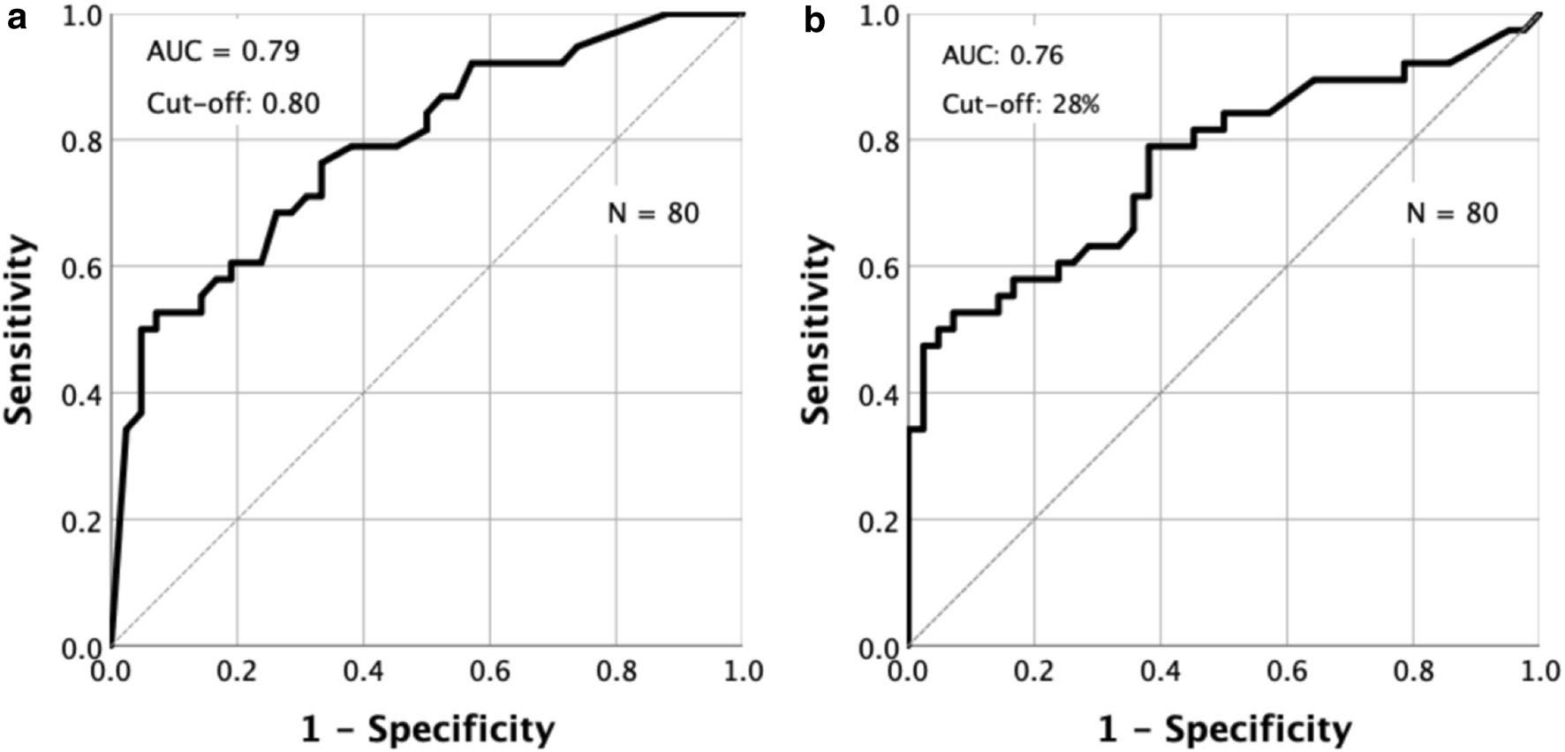
Receiver operating characteristics (ROC)-curves showing the diagnostic ability of the video head impulse test. Receiver operating characteristics (ROC)-curves showing the sensitivity plotted against 1-specificity for different video head impulse test cut-offs, using caloric irrigation derived unilateral weakness > 25% to define unilateral vestibular hypofunction. **a** Lateral canal VOR gain of the affected side as test value, the affected side defined as the side with the lowest caloric response. The area under the curve was 0.79 and a cut-off of 0.80 provided the highest discriminative power, with a sensitivity and specificity of 76% and 55%, respectively. **b** Gain asymmetry as test value. The area under the curve was 0.76. A gain asymmetry cut-off of 28% provided the highest discriminative power, with a sensitivity and specificity of 53% and 93%, respectively. ROC, receiver-operating characteristic. VOR, vestibulo-ocular reflex. AUC, area under the curve

Figure 3a depicts a ROC curve showing the ability of different vHIT lateral canal VOR gain cut offs for the affected ear (regardless of saccades) to detect vestibular hypofunction when using UW to define the actual state (UW > 25% = vestibular hypofunction). The area under the curve was 0.79 (95% CI: 0.69–0.89). The optimal cut-off as determined by Youden index was 0.80, providing a vHIT sensitivity of 76% and a specificity of 55%. Figure 3b depicts a ROC curve of different vHIT GA cut-offs to detect vestibular hypofunction, using the same definition of the actual state as above. The area under the curve was 0.76 (95% CI: 0.66–0.87). The optimal cut-off was 28%, providing a sensitivity of 53% and a specificity of 93% versus the caloric test.

## Discussion

### General Considerations

The main findings of this study were the correlations between caloric irrigation and vHIT despite a 10 days time-lag between the investigations and a high specificity (93%) for GA to predict absence of unilateral vestibular hypofunction in acute vertigo patients.

The key question raised by medical professionals diagnosing vertigo patients is whether caloric irrigation can be replaced by vHIT or not. The two tests have inherent differences. The vHIT measures all six semicircular canals; the caloric irrigation measures only the lateral canals but adds visual suppression. Moreover, the stimulation used differs; caloric irrigation uses a temperature gradient to create a non-physiological endolymphatic flow in the lateral semicircular canals lasting for minutes while the vHIT uses rapid head turns which gives rise to a short lasting physiological endolymphatic flow. One consequence of these differences is that the vHIT result is more dependent on the technician´s skill and experience compared with the caloric testing [7, 9, 16–18]. The low sensitivity of vHIT constitutes an uncertainty in peripheral vestibular diagnostics. As the subjects in this study have acute onset vertigo, and as accuracy is likely to depend on diagnosis, a higher sensitivity may be expected from this selected study sample compared with chronic dizziness. In summary, so far there has not been enough clinical evidence to suggest to exclude the caloric test, and nor does this study supply such evidence.

We report a mean VOR gain of around 0.80 in healthy ears and 0.36 in affected ears among participants with vestibular hypofunction (Table 3). To investigate the appropriate cut-off for the vHIT we used ROC statistics and the Youden index, which indicated that the manufacturer pre-determined VOR gain cut-off of 0.80 provided the highest discriminative power also in this cohort. However, since Youden index assumes a disease prevalence of 50%, gives equal weight to both sensitivity and specificity and ignores the economic aspects of diagnostic decisions, this might not be the most useful cut-off in clinical practice [16]. The potential consequences of missed strokes during acute vertigo stroke risk stratification may argue for a lower cut-off with a higher specificity to avoid false positive investigations. One important finding in this dataset was that GA seemed to be the more clinically useful measure to predict absence of unilateral vestibular hypofunction as a ROC- and Youden index-derived GA cut-off of 28% resulted in a very high (93%) specificity, albeit at the cost of a lower sensitivity. The absence of unilateral vestibular hypofunction in acute onset vertigo indicates a need for a more thorough work-up to identify central causes.

There was a dissociation between specificities of vHIT in the four-field table (Table 2) compared with the ROC-curve analysis, even though they were both based on the same dataset. This is explained by the fact that a pathological vHIT in the four-field table was defined as a VOR gain of < 0.8 of the affected side (determined as the side with the weakest caloric response) in combination with pathological ipsilateral catch-up saccades, whilst the ROC-curve was based solely on the VOR gain of the affected side. Basing the vHIT pathological/healthy status solely on VOR gain gave rise to one more true positive (n = 29 vs 28) and three more false positives (n = 19 vs 16) compared with the more conservative method.

### Potential Physiological Explanations of the Dissociation of vHIT and Caloric Irrigation

In a third of our subjects the results from caloric irrigation and vHIT dissociated, which is comparable to previous studies (Table 3). Park et al. [6] reported dissociation of results in 19 of 92 patients (21%) and van Esch et al. [3] in 82 of 324 (25%). The caloric irrigation and vHIT does to some extent provide unique information of the semicircular canal function. This was suggested by Halmagyi et al. who found that 13 out of 14 patients with intractable vertigo or acoustic neurilemmoma, prior to vestibular neurectomy, had unilaterally diminished caloric responses despite normal reactions to head impulses [17]. That the different ways of semicircular canal stimulation is of importance is also supported by a proposed mechanism between the caloric irrigation and vHIT dissociation in Meniere’s disease; i.e. that the hydropic expansion of the semicircular canal duct in Meniere’s disease leads to dissipation of hydrostatic pressure across the cupula during caloric irrigation whilst the VOR function during vHIT remains unaffected [18].

### Limitations of the Study

As per local clinical routine, some patients’ UWs were based on monothermal warm caloric screening test instead of bithermal caloric irrigation. A study by Murnane et al. [19] suggested that normal findings on the monothermal warm caloric screening test has a high accuracy of predicting a normal bithermal caloric irrigation. They found that an upper normal UW limit of 25% generated a 1% false negative rate of monothermal warm caloric screening test as compared with bithermal irrigation. Bush et al. [20] found a negative predictive value of 90% and sensitivity of 87% for monothermal warm caloric screening test as compared to bithermal caloric irrigation and therefore proposed that it might be of good use as caloric screening. On the other hand, a study by Keith et al. [21] found that the UW of bithermal and monothermal caloric irrigation correlated in patients with UW < 15% or > 30%, but found a lack of accuracy of the monothermal test at the border of normal and abnormal vestibular function. The authors suggested that the poor performance of the monothermal warm caloric screening test in their dataset with a false negative rate of 29% indicated that it cannot replace the bithermal caloric irrigation. A systematic review by Adams et al. [22] supports these findings and in agreement with Shepard and Jacobsen [23] concludes that bithermal caloric irrigation is preferred whenever possible. Our data with slightly stronger correlations between vHIT and bithermal caloric irrigation data compared with monothermal caloric irrigation data support this notion. Another key limitation of this study worth considering is the 10 days time-lag between vHIT and caloric irrigation (Table 1). Ideally, these would have been performed on the same day. The study participants were selected from a prospective observational study aimed to analyze the diagnostic yield of neuro-otological examination for stroke risk differentiation during acute onset vertigo. This entailed that other, clinically more relevant diagnostic procedures and treatments were prioritized, leading to a delay of caloric irrigation and possibly an under-estimation of the sensitivity of the vHIT due to vestibular disorder recovery.

## Conclusion

In this study on acute vertigo patients the results from vHIT correlated with those from caloric irrigation, still neither test seem to have the accuracy to replace the other. GA appears as an attractive measure in acute vertigo as the high specificity can be used to identify those with a substantial probability of normal vestibular function in need of more comprehensive work-up for central causes.

## Data Availability

Source data is available for sharing upon valid request to the corresponding author.
